# Migration status and urologic health inequities: a scoping review

**DOI:** 10.1097/MOU.0000000000001412

**Published:** 2026-05-23

**Authors:** Kimia Goodarzi, Marcin Miszczyk, Pierre I. Karakiewicz, Leonardo O. Reis, Shahrokh F. Shariat

**Affiliations:** a Department of Urology, Comprehensive Cancer Center, Medical University of Vienna, Vienna, Austria; b Collegium Medicum – Faculty of Medicine, WSB University, Dąbrowa Górnicza, Poland; c Cancer Prognostics and Health Outcomes Unit, Division of Urology, University of Montréal Health Center, Montréal, Québec, Canada; d INCT UroGen, National Institute of Science, Technology and Innovation in Genitourinary Cancer (INCT), Campinas; e Immuno-Oncology Institute, School of Life Sciences, Pontifical Catholic University of Campinas, PUC-Campinas, Campinas; f UroScience, School of Medical Sciences, Unicamp, Campinas, São Paulo, Brazil; g Department of Urology, Motol University Hospital and Second Faculty of Medicine, Charles University, Prague, Czech; h Department of Urology, Weill Cornell Medical College, New York; i Department of Urology, University of Texas Southwestern, Dallas, Texas, USA; j College of Medical Health Technologies, Al-Turath University, Mansour, Baghdad, Iraq; k Hourani Center for Applied Scientific Research, Al-Ahliyya Amman University, Amman, Jordan; l Karl Landsteiner Institute of Urology and Andrology, Vienna, Austria; m Institute for Urology and Reproductive Health, I.M. Sechenov First Moscow State Medical University, Moscow, Russia

**Keywords:** health disparities, healthcare access, migrant health, migration, urology

## Abstract

**Purpose of review:**

Migration is an increasingly important determinant of health and healthcare access. Migrants often face language barriers, socioeconomic vulnerability, administrative obstacles, and reduced access to preventive and specialist services. This review summarizes current evidence on migration-related inequities in urologic care.

**Recent findings:**

Available evidence suggests that migrant populations experience disparities across the continuum of urologic care, including screening, diagnostic work-up, access to specialist evaluation, treatment receipt, and follow-up. In urologic oncology, migrants are less likely to participate in screening programs and may present with more advanced disease. Structural barriers, language discordance, low health literacy, cultural beliefs, medical mistrust, and insurance or legal insecurity all contribute to delayed care. Similar inequities are seen in nonmalignant urologic conditions, including lower urinary tract symptoms, overactive bladder, urinary incontinence, and nephrolithiasis, where symptoms are often underreported and undertreated.

**Summary:**

Migration is an important but under-studied determinant of inequity in urologic care. The current literature remains heterogeneous and often fails to distinguish between migration status and race or ethnicity. Future research should use standardized migration-related variables and focus on actionable strategies to improve equitable access, communication, early diagnosis, and high-quality treatment for migrant populations.

## INTRODUCTION

International migration is a major and growing global phenomenon. More than 280 million people currently live outside their country of birth, and migrant populations now form an essential part of healthcare systems and patient populations worldwide [1,2]. Yet, migrants remain disproportionately exposed to barriers in accessing timely, appropriate, and high-quality care [2,3^▪^]. These barriers include administrative complexity, uncertain legal status, inadequate insurance coverage, financial hardship, language discordance, limited health literacy, discrimination, and mistrust of institutions [3^▪^,^4^,^5^].

In healthcare, inequities and disparities refer to avoidable, systematic, and unjust differences in disease burden, access to care, treatment, and outcomes between groups [6]. In urology, such disparities may affect patients with urologic malignancies, lower urinary tract symptoms, overactive bladder, nephrolithiasis, urinary incontinence, and other chronic conditions [7,8,9^▪▪^,^10^,^11^]. Delays in diagnosis and treatment may lead to disease progression, greater symptom burden, avoidable complications, lower quality of life, and worse survival [8,9^▪▪^,^12^,^13^^▪▪^].

Although disparities in urologic care are increasingly recognized, migration status remains insufficiently studied as an independent determinant [6,9^▪▪^,^14^]. Many reports conflate migrant status with race or ethnicity, making it difficult to isolate the role of language, legal vulnerability, acculturation, insurance, country of origin, and duration of residence. This review summarizes current evidence on how migration-related factors influence access to, quality of, and outcomes in urologic care, and highlights strategies to reduce these inequities. 

**Box 1:**
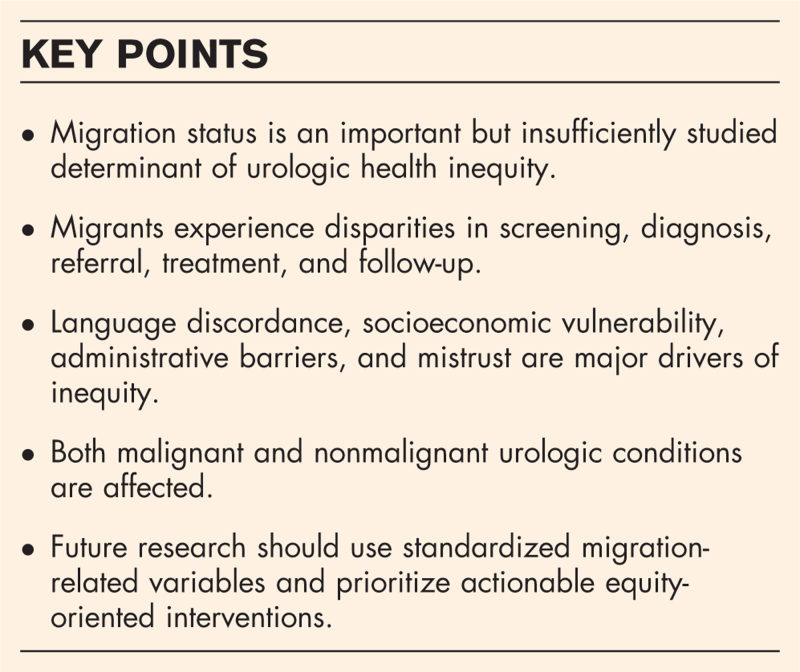
no caption available

## METHODS

This scoping review was conducted in March 2026 to map the existing literature on migration and health disparities in patients requiring urologic care. Literature search was performed in PubMed/MEDLINE, Embase, and Scopus using controlled vocabulary terms and keywords related to migration and urologic health, including “migrant,” “immigrant,” “refugee,” “asylum seeker,” and “migration,” combined with “urology,” “urologic disorders”, “urologic cancer,” “prostate cancer,” “bladder cancer,” “kidney cancer,” “nephrolithiasis,” “kidney stone,” and “lower urinary tract symptoms”. Articles were limited to those published in English.

### Migration as a determinant of urologic health

Migration influences health through a combination of social, structural, and individual-level mechanisms [2,3^▪^]. Among the most important migration-related variables are immigration status, country of origin, duration of residence in the host country, age at migration, proficiency in the local language, educational attainment, employment, and insurance coverage. These variables interact with healthcare systems and shape patterns of healthcare utilization, preventive care uptake, symptom reporting, and treatment adherence [2,3^▪^,^5^,^15^].

Socioeconomic disadvantage is a major contributor to inequity. Migrants are more likely to experience unstable employment, lower income, underinsurance, and housing insecurity. These factors can directly limit access to screening, specialist consultation, diagnostic testing, and definitive treatment [3^▪^,^5^,^9^^▪▪^]. In prostate cancer, for example, lower income and lower insurance coverage are associated with lower rates of prostate-specific antigen (PSA) testing and lower likelihood of receiving definitive local therapy [9^▪▪^,^16^,^17^].

Language is another central driver of inequity. Language discordance can impair history taking, informed consent, shared decision-making, and understanding of treatment options and follow-up instructions. Limited language proficiency is associated with delays in diagnosis, reduced treatment adherence, greater dissatisfaction with care, and poorer outcomes [18^▪^,^19^]. Migrants who are proficient in the host-country language generally navigate healthcare systems more effectively and are more likely to seek preventive and specialist care [3^▪^].

Acculturation and familiarity with the healthcare system also matter. Length of stay in the host country often influences screening participation, symptom recognition, trust in institutions, and comfort engaging with medical professionals [3^▪^,20^▪^]. Newly arrived migrants may be especially vulnerable because they are less familiar with referral pathways, entitlements, and available services.

### Disparities in urologic malignancies

#### Prostate cancer

Among urologic malignancies, prostate cancer is the best studied in relation to migration-related inequity. Existing evidence suggests that migrant men are less likely to participate in PSA screening and may present with more advanced disease [13^▪▪^,^21^]. Lower uptake of screening appears to be driven by limited awareness, language barriers, reduced access to primary care, uncertainty about the value of testing, and culturally shaped beliefs regarding masculinity, sexuality, and cancer [22,23,24].

Several qualitative studies have shown that fear of diagnosis [25,26], misconceptions regarding prostate cancer [25,27], stigma around rectal examination, concerns about sexual function [26–28], and distrust of the healthcare system may all discourage screening [29]. Some migrant groups also show greater reliance on complementary or alternative medicine, partly reflecting barriers to conventional care and high out-of-pocket costs [30].

The consequences are important. Reduced participation in early detection may contribute to delayed diagnosis, higher-risk disease at presentation, and lower likelihood of curative treatment [13^▪▪^]. Recent evidence from broader cancer disparities research also suggests that migrants are less likely to be diagnosed at an early stage and more likely to experience avoidable delays in care [6,13^▪▪^,^22^].

#### Bladder, kidney, and testicular cancer

The literature is far more limited for bladder, kidney, and testicular cancer, but similar mechanisms likely apply. Lower awareness of warning symptoms, delayed specialist referral, poor continuity of care, and reduced access to high-volume centers may disadvantage migrant patients [6,9^▪▪^,^13^^▪▪^]. For bladder cancer specifically, migrant populations from endemic regions may also carry distinct exposure profiles, including schistosomiasis-related risk, which requires culturally and geographically informed assessment [22]. Additionally, exposure to aristolochic acid and arsenic, usually acquired through herbal remedies, contaminated water, or food, constitutes a significant risk factor associated with urothelial carcinoma in migrant populations [31–33].

For kidney and testicular cancers, evidence remains sparse, but structural barriers, delayed presentation, and unequal access to advanced diagnostics and multimodal care are likely to contribute to differences in outcomes [9^▪▪^]. Overall, the field still lacks robust migration-specific analyses across the major urologic cancers.

#### Disparities in nonmalignant urologic conditions

Migration-related inequities are not limited to oncology. Nonmalignant urologic conditions such as overactive bladder, urinary incontinence, lower urinary tract symptoms, and nephrolithiasis are also affected [7,8,10,11,34].

These disorders are common and often profoundly impair quality of life, yet symptoms may be underreported among migrants because of embarrassment, cultural stigma, normalization of symptoms, or lack of awareness that effective treatment exists. Religious or cultural norms may additionally shape symptom disclosure, especially for urinary leakage, sexual symptoms, or pelvic complaints [7,8,11,34].

In nephrolithiasis, environmental change after migration may play a role. Dietary transition, urbanization, occupational exposure, hydration habits, and altered lifestyle patterns after migration may contribute to stone risk. Evidence from population-based cohorts suggests that first-generation immigrants may show different stone incidence patterns than second-generation populations, supporting the relevance of environmental rather than purely genetic factors [10].

Importantly, underdiagnosis and undertreatment of benign urologic conditions can still result in major morbidity, including chronic pain, recurrent emergency visits, lower work productivity, sleep disturbance, social withdrawal, and impaired mental well-being.

### Structural and systemic barriers to urologic care

Many healthcare systems remain poorly adapted to the needs of culturally and linguistically diverse populations. For migrant patients, barriers often accumulate across multiple levels.

At the system level, restrictive policies, fragmented referral pathways, interpreter shortages, and complicated administrative procedures can hinder access. Migrants with uncertain legal status may avoid seeking care for fear of deportation, financial burden, or institutional exposure. Even those with formal legal status may remain underinsured or unaware of their entitlements [9^▪▪^,^13^^▪▪^,20^▪^,^35^].

At the provider level, implicit bias and limited cultural competence can adversely affect communication, trust, and therapeutic relationships. Structural racism is rarely addressed explicitly in urologic literature, yet it may profoundly shape the patient experience [36]. Historical mistreatment and broader experiences of discrimination can generate medical mistrust, which reduces healthcare utilization, screening participation, and acceptance of treatment recommendations [9^▪▪^,20^▪^,^29^].

At the patient level, low health literacy, language barriers, competing financial priorities, transport limitations, and lack of social support all reduce care engagement. These challenges are amplified in rural or underserved regions, where access to specialist urologic services may already be limited [3^▪^,^9^^▪▪^,^37^].

Digital health tools and patient-reported outcome measures may also inadvertently widen disparities if they are not culturally and linguistically adapted. Even professionally translated instruments may remain difficult to understand because concepts, phrasing, and culturally sensitive items do not transfer easily across populations. Recent work suggests that immigrant background may negatively affect compliance with electronic patient-reported outcomes in urothelial cancer care, underscoring the need for language choice, tailored support, and community-sensitive implementation [38,39].

### Strategies to reduce inequities in urology

Reducing migration-related inequities in urologic care requires coordinated action at the research, clinical, health-system, and policy levels.

#### Improve the evidence base

The first priority is better research. Migration status must be defined more rigorously and, when appropriate, separated from race and ethnicity. Studies should routinely capture country of birth, migration category, age at migration, duration of residence, preferred language, need for interpreter services, education, insurance, and legal vulnerability. Prospective cohorts, registry studies, and clinical trials should systematically include migrant populations [6,29].

The field also needs stronger implementation science. It is not enough to describe disparities; research must test interventions that improve screening, referral, adherence, and outcomes.

#### Deliver culturally competent care

Healthcare professionals should be trained in cultural humility, implicit bias, anti-racism, and cross-cultural communication [6,40]. Interpreter services should be integrated into routine care rather than treated as optional. Educational materials should be linguistically accessible and culturally adapted. Bilingual staff and patient navigators can substantially improve communication and continuity [9^▪▪^,^14^].

#### Strengthen community engagement

Community-based interventions are particularly promising. Collaboration with migrant community organizations, community health workers, faith-based networks, and trusted local leaders can improve health literacy, promote screening uptake, reduce stigma, rebuild trust, and support navigation through healthcare systems [6,9^▪▪^,18^▪^].

#### Reduce structural barriers

Policy solutions are also essential. Expanding insurance coverage, simplifying administrative processes, reducing out-of-pocket costs, supporting transport and access to specialist centers, and ensuring safe access to care regardless of immigration status are all likely to improve equity [9^▪▪^,^15^,^40^,^41^]. In parallel, healthcare leadership should increase workforce diversity and decision-making structures to better reflect the populations served [42].

#### Clinical implications

For practicing urologists, migration status should be recognized as clinically relevant. It may affect how patients access care, understand risk, communicate symptoms, engage in screening, interpret recommendations, and adhere to follow-up.

Clinicians should maintain a low threshold for identifying language needs, assessing health literacy, and clarifying barriers to follow-up. They should use trained interpreters where needed, avoid relying on family members for sensitive discussions, and adapt counseling to the patient's cultural and linguistic context.

In oncology, particular attention should be paid to delayed presentation, lower screening uptake, and risk of fragmented care. In benign conditions, clinicians should be aware that symptom burden may be substantial even when complaints are initially minimized. Culturally safe, respectful communication is not an adjunct to good care; it is part of good care.

## CONCLUSION

Migration is an important yet underrecognized determinant of inequity in urologic care. Available evidence suggests that migrant populations may face disadvantages across the full continuum of care, from prevention and early detection to diagnosis, treatment, follow-up, and survivorship. These inequities arise from an interplay of socioeconomic vulnerability, language barriers, administrative complexity, cultural factors, mistrust, and insufficiently adapted healthcare systems.

Despite the clinical relevance of this topic, the literature remains limited, heterogeneous, and methodologically inconsistent. Many studies fail to distinguish migration-related determinants from broader racial or ethnic categories, and key migration variables are often missing.

Future work should adopt standardized migration-related definitions, strengthen inclusion of migrant populations in urologic research, and prioritize interventions that are practical, scalable, and culturally responsive. Addressing these gaps will be essential to improve equitable access to high-quality urologic care.

## Acknowledgements

*None.*

### Financial support and sponsorship

*None.*

### Conflicts of interest

*There are no conflicts of interest.*

## REFERENCES AND RECOMMENDED READING

Papers of particular interest, published within the annual period of review, have been highlighted as:

▪ of special interest
▪▪ of outstanding interest
